# Low-cost fully additively manufactured passive microwave components exploiting available 3D flexibility

**DOI:** 10.1038/s41598-023-30163-4

**Published:** 2023-02-18

**Authors:** Ilona Piekarz, Krzysztof Wincza, Slawomir Gruszczynski, Jakub Sorocki

**Affiliations:** Institute of Electronics, AGH University of Science and Technology, Kraków, Poland

**Keywords:** Electrical and electronic engineering, Mechanical engineering

## Abstract

This paper presents a novel technique for low-cost realization of fully additively manufactured passive microwave components through Stereolithography and selective metallization. The introduction of the third dimension (Z-axis) allows to improve parameters of the circuits (impedance match, isolation, etc.) by realization of variable thickness air layers underneath the circuits’ patterns, which additionally lower the overall circuit losses caused by 3D printing material dielectric properties. Realization of a directional coupler, a low-pass filter, and a patch antenna utilizing the proposed manufacturing technique is discussed in detail and experimentally validated. The obtained measurement results for circuit demonstrators operating within the centimeter frequency range prove the benefits and performance of the introduced technique.

## Introduction

The recently increasing growth of the consumer electronics market, especially smart electronics, in which wireless communication network plays an important role requires new solutions enabling the integration of as many electronics modules in one device as possible. One of the most important parts of these modules is the RF front-end, which allows them to perform wirelessly. To follow the trend of highly integrated and miniaturized devices, high-frequency electronic devices need to feature compact size, lightweight as well as reduced waste, and considerable cost-saving in high-volume production. Additive Manufacturing (AM) is the technology, which can be used to achieve the aforementioned properties of RF electronics.

Initially, AM technology was adopted for rapid prototyping to test the design before the final product development, however recently evolved towards end-use components^1–5^. This is due to the significant improvements in the manufacturing equipment and material engineering, but also due to the design flexibility it provides. AM technology was successfully utilized for the realization of a wide variety of passive microwave devices, including ones being three-dimensional curvy structure^3^. In^4,6–8^ it was shown, that 3D printing technology together with appropriate metallization technique can be utilized for the realization of waveguiding structures, which can feature lower losses than SIW structures, in which an electromagnetic wave fully propagates^9–11^ or partially^12^ within the lossy dielectric substrate. Another approach for the realization of microwave devices was presented in^13–15^, where AM technology was used as a substitute for relatively expensive CNC milling for the realization of the metallic enclosure allowing for the suspension of laminate with the etched pattern being a directional coupler. The great advantage of 3D printing technology is the possibility to create truly three-dimensional structures as compared to two-and-one-half-dimensional techniques such as low-temperature cofired-ceramic (LTCC)^5^.

Among various types of additive manufacturing technologies, vat photopolymerization (VP) is one of the most popular 3D printing technologies that acquired broad attention from a diverse range of fields^16^. VP is a layer-by-layer process in which an ultraviolet (UV) light is focused on a vat of photopolymer resin which solidifies to a given layer pattern. The above process uses either laser or non-laser-based techniques such as Stereolitography (SLA) where laser beam is controlled by a set of moving mirrors or Digital Light Processing (DLP) where a uniform diode-based source is masked by a Liquid Cristal Display (LCD). Recent advances in mechanical engineering have enabled obtaining a printing resolution of tens of micrometer, which is of the same order as Aerosol Jet Printing (AJP)^17,18^ at a fraction of the AJP cost. One of the potential applications for which the VP technology might be well suited is the realization of microwave components^19,20^ when coupled with suitable (non)selective metallization technique. Post-processing of printed parts is necessary as they are made of non-conductive polymer.

In this paper, we present a technique for low-cost realization of additively manufactured microwave components using VP and selective metallization technique. Specifically, we present the ability to realize passive devices such as directional couplers, patch antennas, and filters utilizing strip transmission line techniques and DLP based 3D printing technology. The high electrical performance is obtained by exploiting the third dimension that is used for the realization of variable thickness air layers. In comparison to previously presented solutions^13–15^, no additional microwave laminate is required for the realization of passives and the entire structure can be 3D-printed and metalized, which significantly reduces the cost of production of the microwave elements. The obtained results prove the possibility for realization of fully AM passive microwave components, featuring relatively low-losses and satisfactory electrical properties within the centimeter frequency range.

## Materials and methods

The introduced method of full additive manufacturing of microwave components was validated by exemplary realizations of three different passive elements being basic building blocks for every wireless system i.e., a directional coupler, a low-pass filter, and a patch antenna.

### Coupled-line directional coupler design in stripline technique

**Figure 1:**
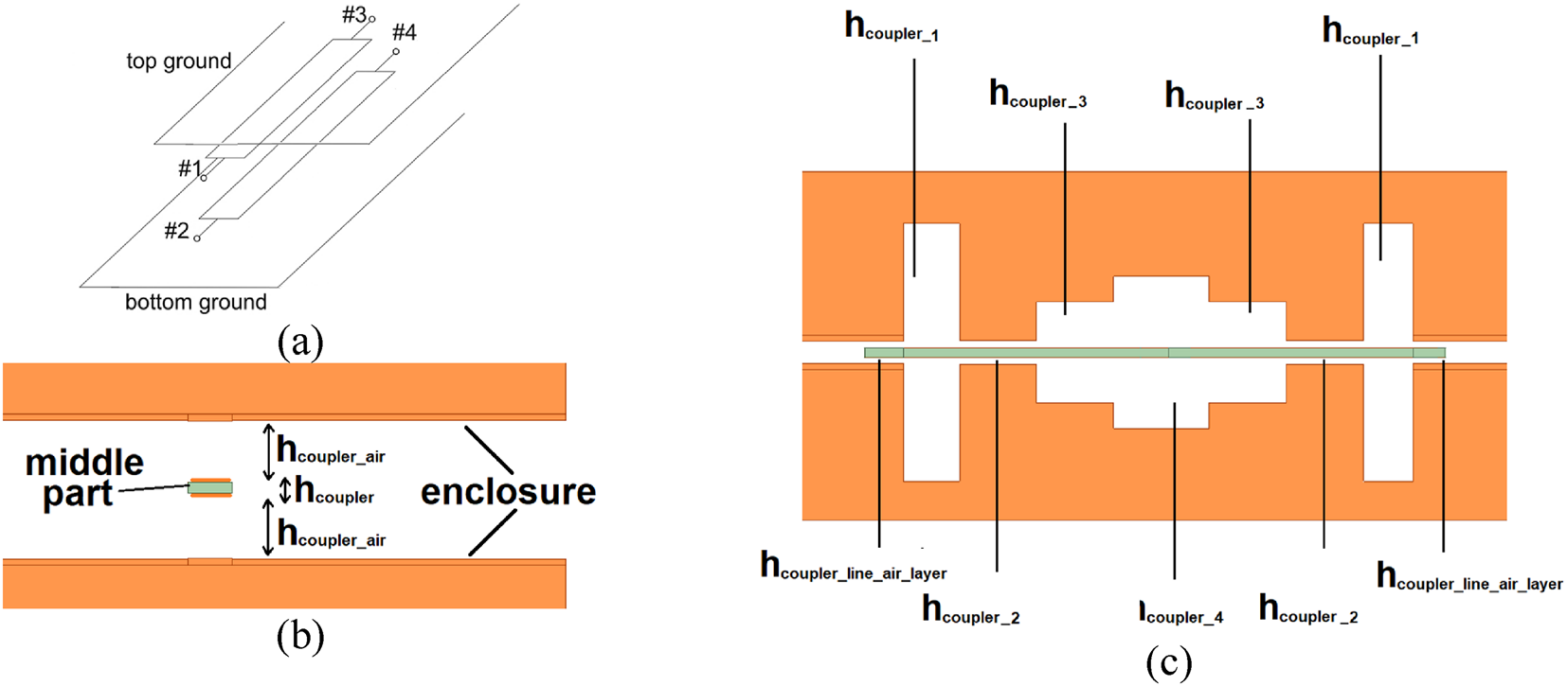
A generic view of a broadside-coupled coupled-line coupler in stripline configuration (**a**), cross-sectional view—the middle part feature width equal to *w*_*coupler*_ (**b**) and side view (**c**). The orange color represents the conductive layer, whereas the green color is the dielectric layer.

Directional couplers are utilized in various applications such as power division and combining networks, power level monitoring circuits, measurement multi-ports, antenna feeding, and beam-forming networks^21^. The coupled-line directional coupler is composed of a section of transmission lines, which are either edge or broadside coupled (see Fig. 1a). Such a configuration allows to obtain broader bandwidth in comparison to branch line couplers^21^ and additionally significantly reduces undesired radiation of the lines. The power delivered to one of the strips is coupled to the other at a level dependent on the coupler’s coupling coefficient, which varies with the distance between the coupled strips and the strips' widths. Frequently used coupling coefficient *k* in directional coupler is *k* = 0.707, namely 3-dB directional coupler, in which the power delivered to port #1 is equally divided between ports #2 and #3, whereas port #4 is isolated (see Fig. 1a).

The directional coupler was designed as a three-part structure (see Fig. 1b) i.e., the upper and lower enclosure, which needs to be metalized, and the middle part is a dielectric layer between the coupled transmission lines connected with input lines. The middle part is hosted by the upper and lower enclosure allowing for the realization of varying air layer suspension. A cross-sectional view of the dielectric stratification is shown in Fig. 1b. In^13–15^ it was shown that this feature can be utilized for partial realization of compensating elements allowing for frequency characteristic improvement of coupled-line couplers. In the proposed realization, due to the full 3D printing of the coupler and the possibility of truly three-dimensional structure utilization, it is possible to fully realize the compensating elements by varying the air layer and the middle part thickness (see Fig. 1c) as to improve impedance matching and isolation.

**Figure 2:**
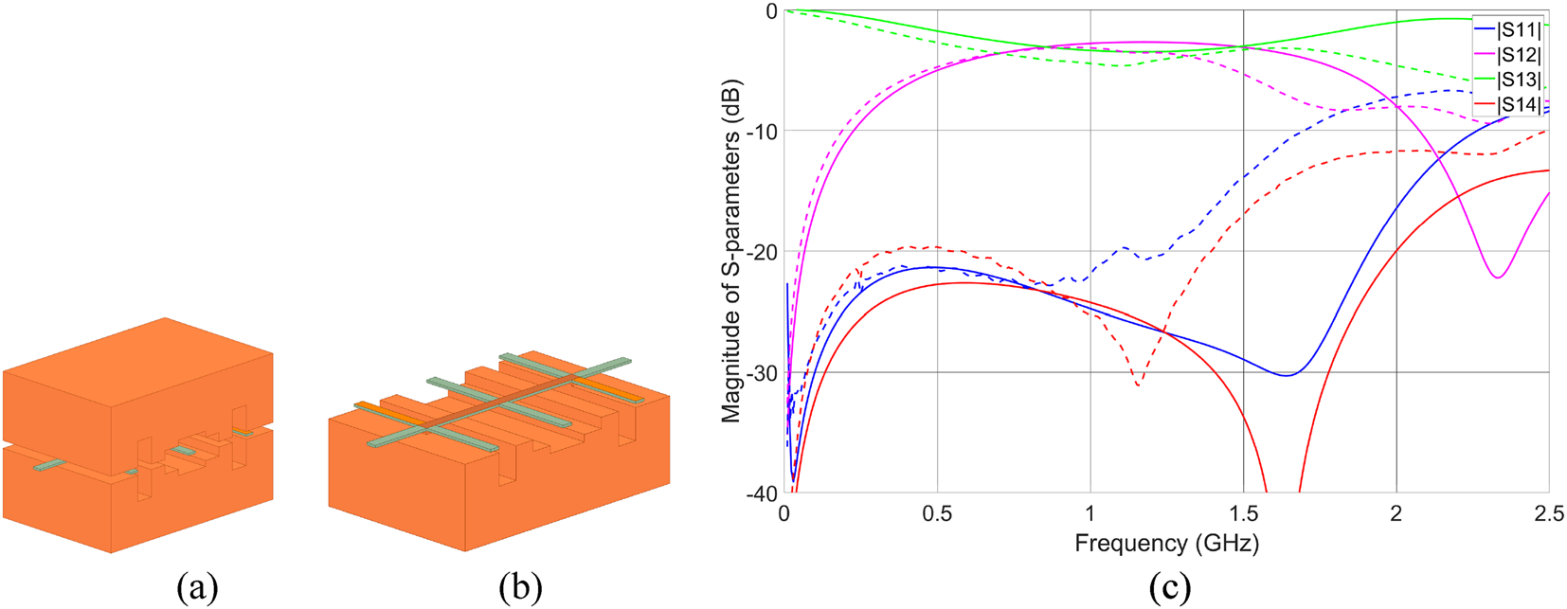
The 3D view on the designed coupler (**a**) and with the top enclosure removed, (**b**) orange color represents the conductive layer, whereas the green color the dielectric layer. The frequency characteristics of the designed coupler: EM simulation results—solid lines and measurement results—dashed lines (**c**).

The initial dielectric stratification was calculated using *Linpar* software^22^ to obtain a 3-dB @ 1 GHz directional coupler having characteristic impedance *Z*_0_ = 50 Ω and to be mechanically rigid when 3D printed. The permittivity and loss tangent of the middle dielectric layer was assumed to be equal to 2.8 and 0.01, respectively. With such assumptions, the middle part width and height were calculated as *w*_*coupler*_ = 2.5 mm and *h*_*coupler*_ = 0.6 mm, respectively, and was suspended over an air layer height *h*_*coupler_air*_ = 1.55 mm (see Fig. 1b). In the next step, the coupled-line section was divided into eight subsections (see Fig. 1c) and for each subsection, the air-layer thickness (*h*_*coupler_air_1*_, *h*_*coupler_air_2*_,* h*_*coupler_air_3*_, *h*_*coupler_air_4*_) was set for further optimization to improve impedance match and isolation characteristics of the designed coupler. Moreover, an air layer height *h*_*coupler_line_air*_ = 0.8 mm was determined using^22^ to realize 50- Ω input transmission lines, for which due to the design simplicity the same width *w*_*_coupler*_ as for the coupled-line section was assumed. Since the transition region between the input lines and the coupled line section introduces parasitic reactances^23^, their influence was compensated by changing the self-capacitance value in this transition region using the air layer thickness change *h*_*coupler_line_air*_*comp*_. Finally, the designed coupler was electromagnetically simulated using *Ansys HFSS* software and four parameters were set for optimization to improve its frequency characteristics i.e., *h*_*coupler_air_1*_, *h*_*coupler_air_2*_,* h*_*coupler_air_3,*_ and *h*_*coupler_line_air*_*comp*_. The final layout of the circuit is presented in Fig. 2a,b, whereas the geometrical parameters found with the optimization procedure are equal: *h*_*coupler_air_1*_ = 8.08 mm, *h*_*coupler_air_2*_ = 0.45 mm, *h*_*coupler_air_3*_ = 2.96 mm, *h*_*coupler_air_4*_ = 4.63 mm, *h*_*coupler_line_air*_*comp*_ = 1.20 mm. Elongation of the middle part visible in Fig. 2a,b is required for further mounting purposes. The simulated frequency characteristics are shown in Fig. 2c. As seen, the circuit features an equal power split at the center frequency of 1.1 GHz with a coupling imbalance equal to 0.4 dB. The isolation and impedance match at the center frequency of operation is better than 26 dB.

### Low-pass stub impedance filter design

**Figure 3:**
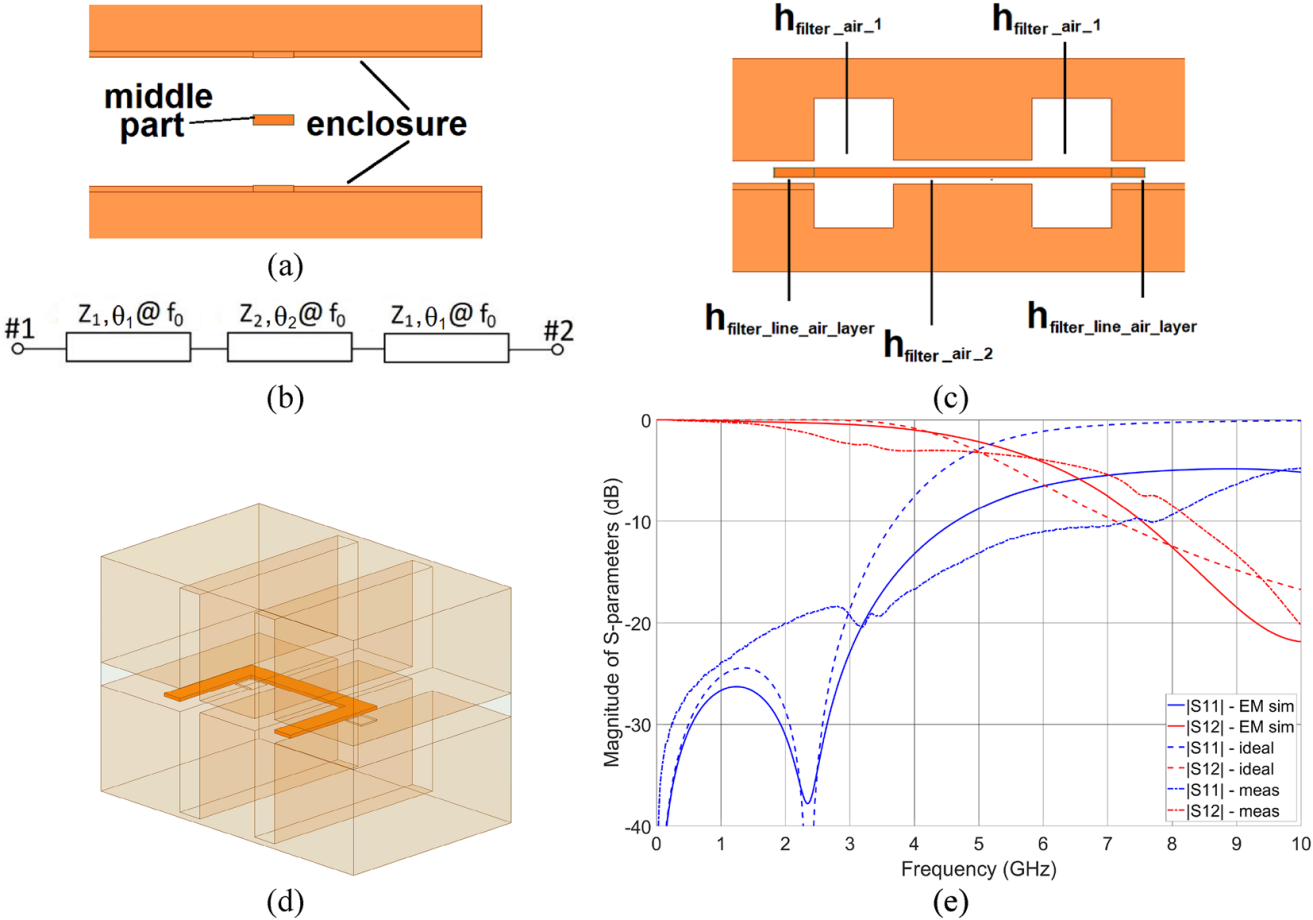
Cross-sectional view—the middle part feature width equal to *w*_*filter*_ (**a**). General schematic of a designed step-impedance low-pass filter (**b**). Side view of the designed filter (**c**). The orange color represents the conductive layer. The 3D view of the designed filter (**d**). The EM calculated frequency characteristics in comparison with the ones obtained for ideal transmission line elements and measurement results (**e**).

Generally, microwave devices fabricated on standard dielectric materials such as laminates suffer from dielectric loss, thus one of the methods allowing for loss reduction is a suspension of a planar circuit above the ground layer^13–15,24^ as to introduce the lossless air layer. The suspended dielectric stratification utilized for the realization of a low-pass filter is shown in Fig. 3a. As seen, similarly as in the case of the coupled-line coupler described in “Coupled-line directional coupler design in stripline technique” section the structure is composed of three parts i.e., top and bottom enclosure along with a middle part which is used for the filter metal pattern design. Nevertheless, in this case, the entire middle part is metalized (without elongation of the middle part required for the assembly process). In such a case, the middle part dielectric loss tangent does not influence the entire circuit losses and the EM waves fully propagate in a lossless air layer in contrary to realizations presented in^13–15,24^.

For the realization of a low-pass filter, a 3rd-order stepped impedance topology shown schematically in Fig. 3b was utilized. As seen the filter was realized using three transmission line section, the two of which feature characteristic impedance *Z*_1_ = 200 Ω and the electrical length *θ*_1_ = 7° @ *f*_0_, whereas the third one features characteristic impedance *Z*_2_ = 20 Ω and the electrical length *θ*_2_ = 18° @ *f*_0_. Moreover, the Chebyshev transfer function was used to approximate the filter response to provide maximum stopband attenuation for a given filter degree, assuming a 0.1 dB ripple in the pass-band of the filter. The filter was designed to have a cut-off frequency of *f*_*T*_ = 5 GHz. The same strip width *w*_*filter*_ = 2 mm was assumed across all sections with a varying air layer thickness (*h*_*filter_air_1*_, *h*_*filter_air_2*_) to obtain an appropriate impedance value, as can be seen in Fig. 3c. Moreover, an air layer height *h*_*filter_line_air*_ = 0.96 mm was determined using^22^ to realize 50- Ω input transmission lines, for which due to the design simplicity the same width *w*_*_filter*_ as for the filter was assumed. The transition region between the input lines and the filter was compensated similarly as it was presented in “Coupled-line directional coupler design in stripline technique” section using the air layer thickness change *h*_*filter_line_air*_*comp*_. Finally, the designed filter was electromagnetically simulated using *Ansys HFSS* software and three parameters were set for optimization to improve its frequency characteristics i.e., *h*_*filter_air_1*_, *h*_*filter_air_2,*_ and *h*_*filter_line_air*_*comp*_. The initial values for *h*_*filter_air_1*_ and *h*_*filter_air_2*_ were obtained using^22^. The final layout of the circuit is presented in Fig. 3d, for which the geometrical parameters found with optimization procedure are as follows: *h*_*filter_air_1*_ = 10.50 mm, *h*_*filter_air_2*_ = 0.42 mm_*,*_ and *h*_*filter_line_air*_*comp*_ = 0.96 mm. Similarly, as in the case of the coupled-line coupler, an elongation of the middle part was utilized, however, it is not visible in Fig. 3d. The simulated frequency characteristics are shown in Fig. 3e together with the ones obtained for ideal transmission line elements. As seen, the obtained frequency characteristics are in good agreement with the ones calculated for an ideal filter.

### Patch antenna design

**Figure 4:**
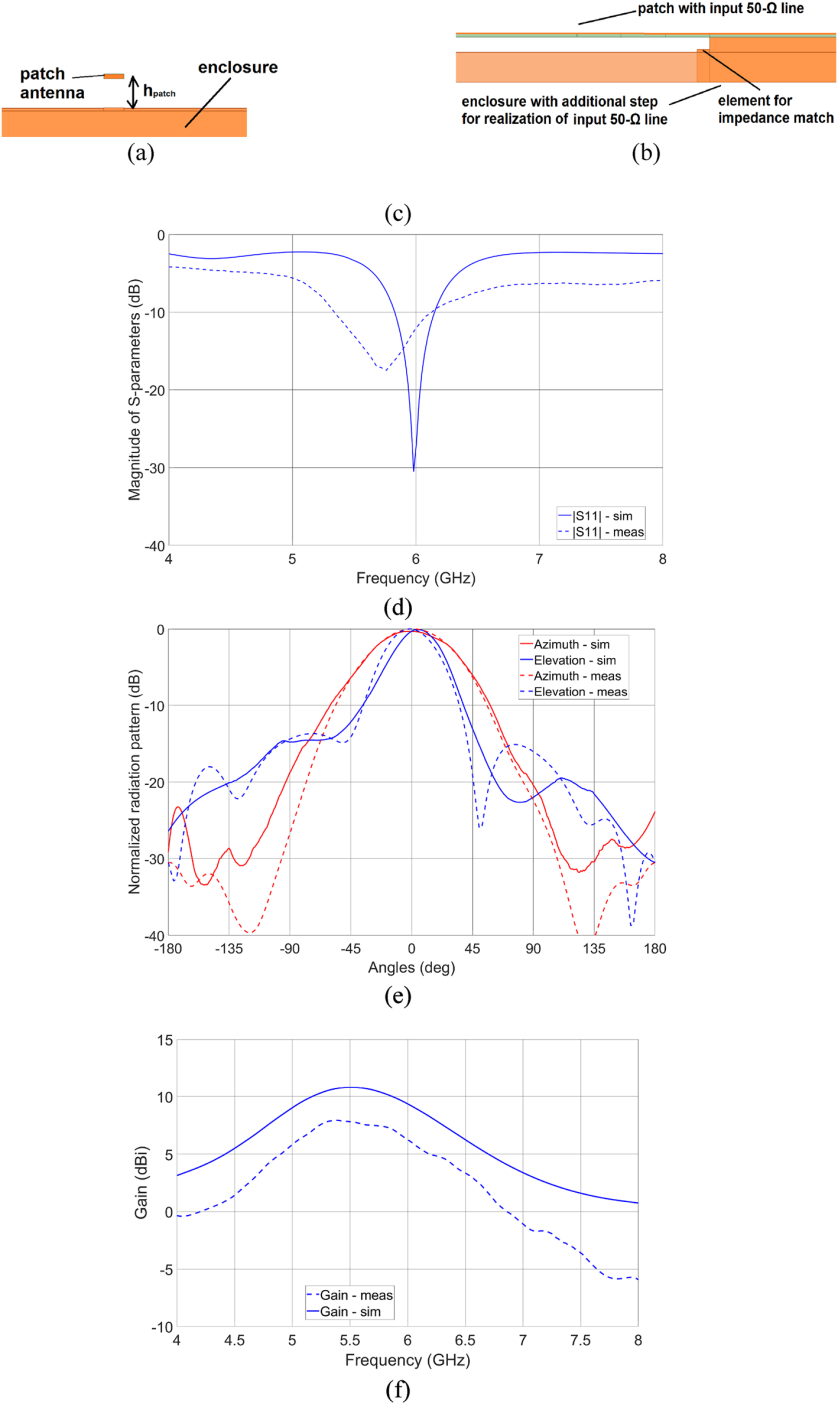
Cross-sectional view (**a**), side view (**b**), and 3D view (**c**) on the patch antenna structure. Impedance match—simulation and measurement results (**d**) and radiation pattern in two main cross-sections: Elevation (blue line) and Azimuth (red line)—simulation and measurement results (**e**). Measured (dotted lines) and simulated (solid lines) gain of the designed antenna (**f**).

Finally, an exemplary microstrip patch antenna was designed in a dielectric structure presented in Fig. 4a to operate at the center frequency *f*_0_ = 6 GHz. The patch is suspended over an air layer having a thickness *h*_*patch*_ = 2.51 mm. Similarly, as for the directional coupler design, the permittivity and loss tangent of the middle dielectric layer were assumed to be equal to 2.8 and 0.01, respectively. The 50-Ω input transmission line was designed in a microstrip structure utilizing third-dimensional design flexibility, as presented in the side view of the structure in Fig. 4b. To obtain impedance match to 50-Ω input transmission line instead of inset-fed topology, an air layer thickness tunning underneath the area of signal line connection was utilized (see Fig. 4b). Final layout of the patch antenna is presented in Fig. 4c with main geometrical parameters marked, where elongation of the middle dielectric part required for mounting purpose is also visible. The geometry parameters are as follows: *patch_x* = 11.92 mm, *patch_y* = 12.24 mm, *w*_*enl*_ = 8.00 mm, *h*_*diel*_ = 0.60 mm, *w*_*comp*_ = 2.25 mm, *l*_*comp*_ = 5.62 mm, *h*_*comp*_ = 2.06 mm. The antenna was simulated using Ansys HFSS software and the obtained impedance match, as well as radiation patterns in azimuth and elevation, are visible in Fig. 4d,e, respectively. The simulated gain at the center frequency of operation *f*_0_ = 6 GHz equals 9.4 dBi (see Fig. 4f).

### Fabrication

**Figure 5:**
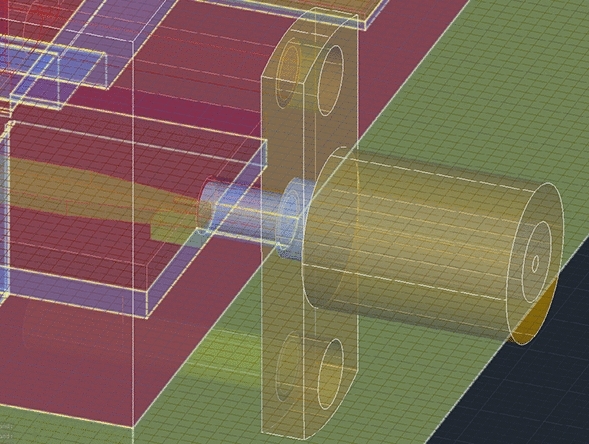
Mount design for panel mounted SMA connectors. Pressure-assisted contact of the center pint with transmission line metal is ensured as well as the ground tabs so as not to require neither soldering nor gluing. This is ensured by proper locating of the connector and using a set of screws, respectively.

Demonstrators of all the above circuits were fabricated using the following procedure. First, all printable models were designed using 3D modeling CAD software based on the primitives exported out of *Ansys HFSS* simulation software. The final models of the directional coupler and filter were created out of three pieces i.e., top, and bottom enclosures and the middle inset, whereas the model of the printed antenna was composed of two parts i.e., the bottom ground plane and the patch element. The three- and two-piece elements are intended to comprise self-enclosed circuits. For that reason, the mechanical and electrical features are complementary to each other. To ensure mechanical strength and mounting points for the top and bottom halves of the enclosure as well as to provide a low-inductance connection between top and bottom ground planes, large surface area pads were designed together with a set of lego-like tightly fitted post and hole elements. Each circuit was fitted with a panel-type mount for the SMA connector, so the center pin with dielectric around can be slid into a dedicated hole while ground metal sides screwed to the enclosure. The center pin contacts the transmission line strip through the mount applying light pressure as shown in Fig. 5, so no soldering or gluing is required.

**Figure 6:**
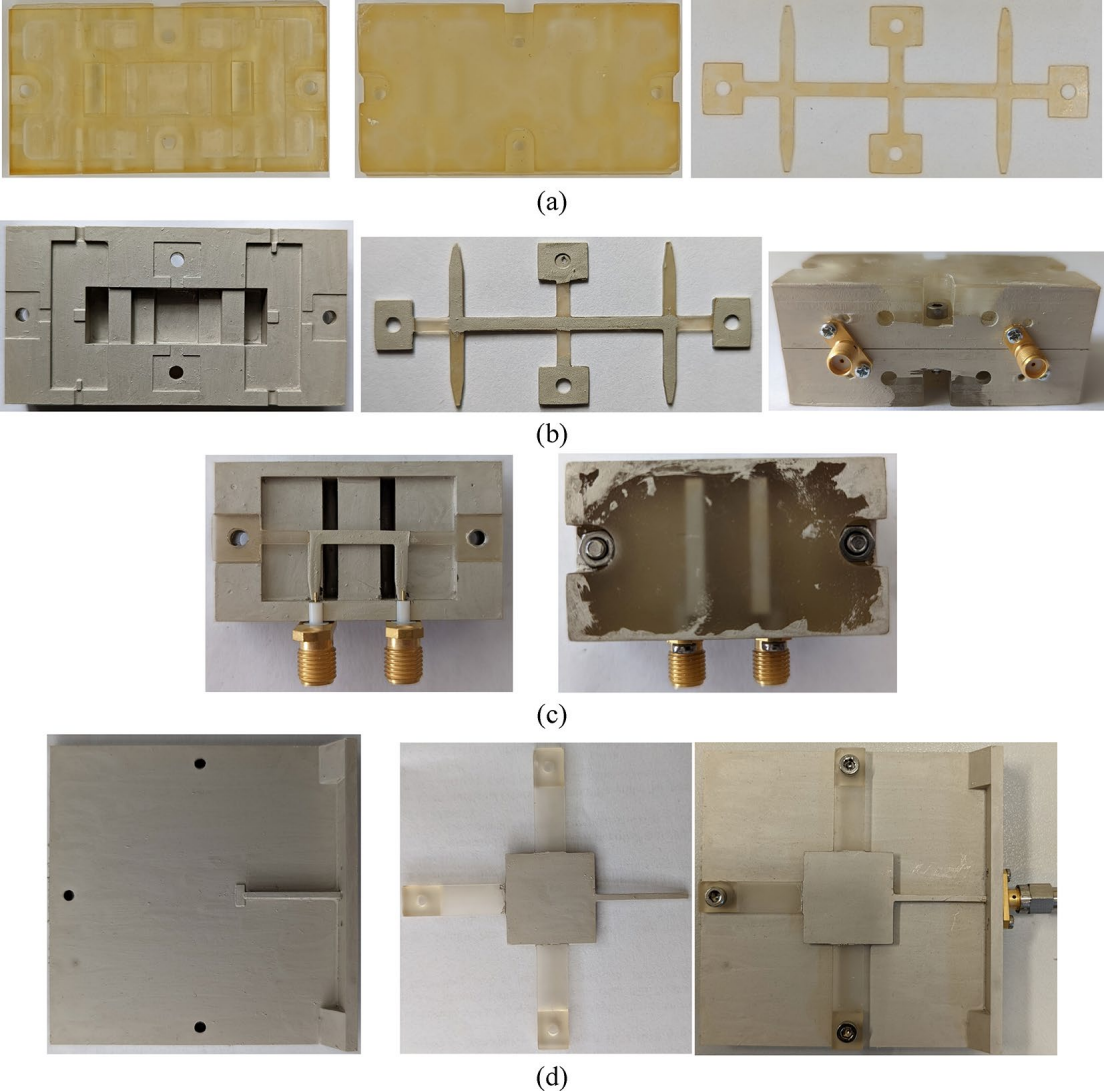
Fabrication steps of circuit demonstrators: printed and cured plastic top/bottom and middle pieces of the directional coupler (**a**), bottom, middle and entirely assembled pieces of directional coupler after metallization process (**b**). A constant strip width coupled section and corresponding variable air thickness enclosure are visible with dielectric mechanical supports as well. Top/bottom and middle pieces of the low-pass filter as well as entirely assembled circuit after metallization process (**c**). Bottom and middle piece as well as entirely assembled patch antenna after metallization process (**d**).

All the elements were prepared, and 3D printed using the Prusa Research hardware-software ecosystem for hobbyists. The Original Prusa SL1S printer was used, which is an DLP type printer (Masked Stereolitography MSLA as described in specification) utilizing a high-resolution LCD panel having a pixel size of 47 µm (50 µm XY-resolution) and a UV LED panel to cure thin layers of resin. A high-quality UV photosensitive Prusa Polymers Transparent Tough resin was chosen as a base material. The material is expected to exhibit very low post-curing linear shrinkage in the range of 1% that should not affect either geometry or dielectric-stack up. The CAD software exported models were sliced using the Prusa Slic3r software at 50 µm layer height being aligned with an integer multiple to the minimum step in the Z-axis of the model (here 0.3 mm) to generate the printer commands files. The default curing profile for the tough resin was used. Models’ parts were placed directly on the build platform to ensure maximally flat and even X–Y surfaces which are crucial for achieving good effective conductivity of the ground plane. This way the surface of the finished print is only affected by the size and construction of the LCD. The UV light during the curing process is being shuttered by the pixel edges resulting in uneven exposure of the resin and thus peaks and valleys reflecting the panel arrangement. The printed units were post-processed using the Original Prusa CM1 curing-washing machine by firstly washing in an isopropyl alcohol to remove excess of uncured resin, then pre-dried using compressed air for clean surfaces, dried in a heated chamber and finally UV cured (as the 3D printer only pre-cures the resin); processing as recommended in resin`s datasheet. Photographs of the 3D printed parts assembly of the directional coupler, filter, and patch antenna are shown in Fig. 6.

At the second stage of fabrication, the printed parts were selectively metalized. The MG Chemicals 842WB—Super Shield Water Based Silver Conductive Coating paint was used targeting at least the recommended 25 µm film thickness (surface resistance of 0.02 Ω/sq), by applying 2 coats with a paintbrush (one coat yields thickness in the range of tens of microns). Paint coverage and film uniformity was inspected visually under bright light (transparent resin was used, apart from other reasons, to simplify the metal film properties inspection) after each coat. The proposed metallization technique has been shown to yield relatively good results even for circuits operating within 24 GHz band^25^. A patterned part was obtained by using masking tape and paintbrush. Photographs of the metalized 3D printed elements of microwave passives are shown in Fig. 6b–d. As seen, the directional coupler and filter middle inset, as well as a patch antenna, were selectively metalized. After manufacturing all the parts, the circuits were assembled and SMA connectors were added. All of the parts were snapped together using the lego-like process. Moreover, a set of screws was used to tighten all the press-contact parts.

## Results

**Figure 7:**
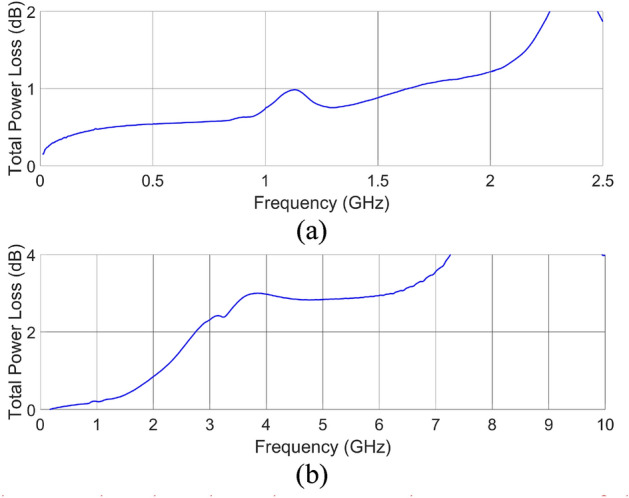
Calculated total power loss based on the measured S-parameters of the fabricated unit of a directional coupler (**a**) and a low-pass filter (**b**).

The usability of the proposed low-cost additively manufacturing approach towards the realization of microwave components was evaluated based on the measurement results of the developed directional coupler, low-pass filter, and patch antenna. The end goal here is to verify if the 3D printing using a low-cost Stereolithography printer together with selective metallization using silver-based paste having surface resistance @ 25 µm 0.02 Ω/sq allows for realization of microwave circuits operating in the frequency range of a few GHz, featuring total power losses at an acceptable level. First, the scattering parameters of the manufactured circuits were measured. The Agilent PNA Network Analyzer N5227A calibrated using SOLT standards with the reference plane set at the SMA connectors plane was used. The measured S-parameters are presented in Figs. 2c, 3e and 4d for directional coupler, low-pass filter, and patch antenna, respectively, whereas the calculated total power losses of coupler and filter are presented in Fig. 7a,b. The normalized radiation patterns of the manufactured patch antenna were measured in two main cross-sections at the frequency *f*_0_ = 5.8 GHz, where the best impedance match was obtained, and the results are shown in Fig. 4e. The impedance match of the manufactured directional coupler at the center frequency of operation is better than − 21 dB, whereas the isolation is better than − 25 dB. The coupler features an equal power split at − 3.76 dB with a coupling imbalance equal to 0.65 dB Total power loss equals roughly 0.8 dB at center frequency. The filter`s transmission and reflection crosses at 8 GHz while its total power loss 4 dB up to that frequency. The printed antenna impedance match is better than − 17. 5 dB at the center frequency of 5.8 GHz, at which the gain is equal 7.4 dBi (see Fig. 4f). The measured gain of the realized antenna is 2 dB lower at the frequencies, where best impedance match was obtained, what can be caused by slightly higher loss tangent of the dielectric material than the assumed one and uneven metal layer on the radiating patch. The 3-dB beamwidths of the measured radiation patterns equal 40 deg in elevation and 61° in azimuth, thus are 8° and 11° greater in comparison to the radiation patterns obtained in simulations. The calculated radiation efficiency for the manufactured antenna at *f*_0_ = 5.8 GHz is equal 40.2%, whereas the expected one calculated based on simulation results at *f*_0_ = 6 GHz is equal 42%. As seen, the obtained measurement results prove the usefulness of the presented approach for the low-cost realization of passive microwave circuits using the additive manufacturing technique. The center frequencies of operation of the manufactured directional coupler and antenna shifted down in comparison to the simulated results. This can be caused by the slightly higher permittivity of the material used for 3D printing, than the assumed value, which was determined for another batch^26^.

## Discussion

In this study, we investigated the approach for low-cost realization of passive microwave components using solely additive manufacturing processes. For experimental study, three basic microwave components were designed, manufactured, and measured namely, a coupled-line directional coupler, a low-pass filter, and a patch antenna. A combination of hobbyist-grade DLP (MSLA) type 3D printer and silver paint-based metallization technique were used for fabrication.

One of the often-used schemes for additive manufacturing of microwave components^4,27^ is a two steps process requiring firstly printing the design using dielectric material and secondly deposition of conductive material. Nevertheless, the circuits shown in literature are usually waveguide structures^20,25,27^ or transmission line type circuits, where the circuit mosaic is in a form of a printed circuit board^13–15^, and the additively manufactured part is an enclosure serving as a ground plane/planes for suspended microstrip or stripline technique. In this paper, we present and a PCB-less approach where components are designed in strip transmission line technique with a focus put on exploiting the third geometrical dimension towards high electrical performance as the fabrication employs dielectric 3D printing and selective metallization. Since low-cost resins are usually relatively lossy, the use of suspend technique is proposed to minimize the total loss by introduction of lossless air to the dielectric stack-up. The above is widely used technique in microwave engineering generally implemented using a combination of PCB and CNC milling processes, i.e., the circuit mosaic is realized on laminate (PCB), whereas the metal enclosure is manufactured using CNC milling process. The suspended technique offers many advantages such as: lower losses and high Q-factor of resonators since as indicated, majority of the field is within air; realization of a wide range of transmission line impedance values; possible wideband operation of circuits, etc. Relating the above to the circuits under study, among many dielectric stack-ups, in which directional couplers can be implemented, a suspended stripline structure is very attractive, since it features low complexity (single laminate layer placed in-between two ground planes) and low losses. Nevertheless, the coupled-line couplers designed in such structure exhibit unequal capacitive and inductive coupling coefficients, thus impedance match and isolation of such circuits are deteriorated^28^. To improve their performance, it is required to equalize the coupling coefficients, what can be achieved by adding compensating elements to the coupled line section^28^. In^13–15^ it was shown, that by varying the air-layer thickness it is possible to partially realize the compensating elements. Nevertheless, since the coupled lines were realized on a laminate having specific thickness it was not possible to realize the compensating elements fully. In this paper, no laminate of fixed thickness is used, instead the entire circuit is 3D printed and thus both variable air layer thickness and dielectric layer thickness can be fabricated. As a result, the compensation can be implemented fully by varying aforementioned thicknesses while keeping the conductors (here coupled strips) at fixed width. Such an approach is also significantly more desired from the manufacturing perspective, since the compensating elements are usually fine features within the metal pattern, which would be relatively hard to fabricate using the proposed approach. The use of variable air/dielectric thickness capability can be further extended to e.g., compensate the discontinuity between the 50-Ω input transmission lines and the coupled lines (directional coupler) or between the feed and patch of the patch antenna. In the antenna case, matching usually requires cutting slots that protrude into the patch as to connect the signal line closer to the antenna`s center. Not needing the above makes the manufacturing process more feasible even using hobbyist grade 3D printer as present the paper. From the filter design perspective possibility of utilization of varying air-layer thickness is also very important, since the presented stub impedance filter requires connection of transmission lines featuring significant difference in their widths and lengths (*Z*_1_ = 200 Ω and the electrical length *θ*_1_ = 7° @ *f*_0_, *Z*_2_ = 20 Ω and the electrical length *θ*_2_ = 18° @ *f*_0_—see Fig. 3b) and especially the high impedance, electrically short section would be difficult to 3D print. Such a construction would be also mechanically less durable, than in the case of a uniform, relatively wide, fixed width line that constitutes the filter`s center conductor.

Taking all the above into account, due to the three-dimensional flexibility offered by the 3D printing process it was possible to realize fully additively manufactured microwave passive circuits operating in the low GHz frequency range and featuring low-cost realization and relatively high-performance. Even though there are AM techniques which allow for realization of passive microwave components in one run (dielectric and metal printed at once)^17^, at this stage of technology development they are neither low-cost nor applicable for cm-wave range circuits. The obtained results have proven, that AM technology is disruptive and enabling technology for the realization of microwave devices, which is promising for the next generation of communication systems, where there will be a need for low-cost in high-volume production, lightweight systems with increased power efficiency. Nevertheless, there are still challenges to the use of this technique including efficient and easy to remove masking of the metallic pattern as well as maximization of the metal layer conductivity. The above are subjects of further studies.

## Conclusion

A novel manufacturing scheme allowing for the realization of passive microwave components that employed a variant of Vat Photopolymerization being the DLP (MSLA) technology coupled with selective metallization was proposed. The approach was investigated on an example of basic microwave building blocks such as coupled-line directional coupler, low-pass filter, and patch antenna demonstration of which were designed, manufactured, and characterized. The experimental circuits exhibit satisfactory impedance match and isolation characteristic (directional coupler, filter) or radiation pattern (antenna). The center frequencies of operation of the directional coupler and antenna units shifted down in comparison to the simulated results, what can be attributed to slightly higher than simulation assumed permittivity of the used resin. Moreover, the measured gain of the fabricated antenna is 2 dB lower at the frequencies of best impedance match, what can be attributed to slightly higher than simulation assumed loss tangent of the used dielectric resin along with higher than simulation assumed effective conductivity of the metallization layer. Even though, the experimental results for proof-of-concept circuits confirm the applicability of the presented approach, especially for realization of microwave circuits designed using suspended strip transmission line techniques operating in the low-GHz frequency range. Moreover, it is also seen that the third dimension of geometrical flexibility when enabled by the technology can be leverage for providing circuits performance improvement by realization of necessary compensating elements using a variable thickness air layer underneath the circuits’ patterns. Finally, the study showed that that the proposed approach provides relatively high-performance, lightweight, fully AM fabricated microwave components at low-cost using inexpensive machinery and materials in a compact form factor what is necessary towards mass production.

## Data Availability

The datasets used and/or analysed during the current study available from the corresponding author on reasonable request.
